# Comparative Effectiveness of Chuna Manipulative Therapy for Non-Acute Lower Back Pain: A Multi-Center, Pragmatic, Randomized Controlled Trial

**DOI:** 10.3390/jcm9010144

**Published:** 2020-01-05

**Authors:** Sun-Young Park, Eui-Hyoung Hwang, Jae-Heung Cho, Koh-Woon Kim, In-Hyuk Ha, Me-riong Kim, Kibong Nam, Min ho Lee, Jun-Hwan Lee, Namkwen Kim, Byung-Cheul Shin

**Affiliations:** 1 Department of Korean Rehabilitation Medicine, Pusan National University Korean Medicine Hospital, Yangsan 50612, Korea; shl0305@pusan.ac.kr (S.-Y.P.); taichi@pusan.ac.kr (E.-H.H.); 2 Department of Korean Medicine, School of Korean Medicine, Pusan National University, Yangsan 50612, Korea; 3 Division of Clinical Medicine, School of Korean Medicine, Pusan National University, Yangsan 50612, Korea; 4 Department of Korean Rehabilitation Medicine, Kyung Hee University Korean Medicine Hospital at Gangdong, Seoul 05278, Korea; spinacho@khu.ac.kr (J.-H.C.); omdkimkw@khu.ac.kr (K.-W.K.); 5 Jaseng Spine and Joint Research Institute, Jaseng Medical Foundation, Seoul 06110, Korea; hanihata@jaseng.org; 6 Jaseng Hospital of Korean Medicine, Seoul 06110, Korea; therev@jaseng.co.kr; 7 Mokhuri Neck & Back Hospital, Seoul 06272, Korea; prebong@naver.com (K.N.); benzengine@hanmail.net (M.h.L.); 8 Korean Medicine Life Science, University of Science & Technology (UST), Campus of Korea Institute of Oriental Medicine, Daejeon 34054, Korea; omdjun@kiom.re.kr; 9 Center for Comparative Effectiveness Research & Economic Evaluation in Korean Medicine, Pusan National University, Yangsan 50612, Korea; drkim@pusan.ac.kr

**Keywords:** Chuna manipulative therapy, lower back pain, non-acute, pragmatic randomized controlled trial, comparative effectiveness research, safety

## Abstract

Current evidence on the effectiveness and safety of Chuna manipulative therapy (CMT) for managing non-acute lower back pain (LBP) is insufficient. We investigated the comparative effectiveness and safety of CMT, a Korean style of manipulation, plus usual care (UC) compared to UC alone for non-acute LBP. We conducted a parallel, two-armed, multi-centered, assessor blinded, pragmatic, randomized controlled trial at four major Korean medical hospitals. Overall, 194 patients were randomly allocated to either CMT plus UC (*n* = 97) or UC alone (*n* = 97), for six weeks of treatment and six months follow-up. The primary outcome was measured using the numerical rating scale (NRS) of LBP intensity at 7 weeks. Secondary outcomes included NRS of leg pain, Oswestry Disability Index (ODI) for functional disability, patient global impression of change (PGIC) scale, and safety. A total of 194 patients were included in the intention-to-treat analysis, and 174 patients provided complete data for the primary outcome. At 7 weeks, clinically significant differences between groups were observed in the NRS of LBP (CMT + UC: −3.02 ± 1.72, UC: −1.36 ± 1.75, *p* < 0.001), ODI scores (CMT + UC: −5.65 ± 4.29, UC: −3.72 ± 4.63, *p* = 0.003), NRS of leg pain (CMT + UC: −2.00 ± 2.33, UC: −0.44 ± 1.86, *p* < 0.0001), and PGIC (CMT + UC: −0.28 ± 0.85, UC: 0.01 ± 0.66, *p* = 0.0119). Mild to moderate safety concerns were reported in 21 subjects. CMT plus UC showed higher effectiveness compared to UC alone in patients with non-acute LBP in reducing LBP and leg pain and in improving function with good safety results using a powered sample size and including mid-term follow-up.

## 1. Introduction

Lower back pain (LBP) is a highly common musculoskeletal disorder causing severe pain, increased sick leave, and heightened social costs [1,2]. LBP is estimated to affect approximately 70% to 80% of individuals over the lifespan [3]. Despite extensive global research efforts, non-acute pain remains a challenge for clinicians and society as a substantial socio-economic problem [3]. The latest Clinical Practice Guidelines (CPGs) suggest various LBP treatments, including both invasive and non-invasive therapies such as pharmacological, psychological, physical, manipulative, and educational therapies [4,5]; of these, spinal manipulation has been recommended for chronic, sub-acute, and acute LBP patients with low to moderate quality of evidence [6,7]. There is also a need for appropriate research designs that effectively reflect the person-centered treatment system promoted in manipulative treatments and provide data that can influence policy decisions within the healthcare system.

Chuna manipulative therapy (CMT) is a Korean style of manipulation that encompasses techniques aiming to restore the balance between anatomical structures and function and is performed by health professionals known as Korean medical doctors [7,8]. CMT focuses on meridian theory and meridian muscle concepts not typically used in chiropractic medicine or osteopathic manual medicine [8]. CMT also incorporates imaging analyses in diagnoses.

CMT has high patient satisfaction and several published randomized controlled trials (RCTs) have verified the effectiveness and safety of CMT. However, RCTs to date have limitations regarding study design and sufficient power to adequately evaluate CMT effects and safety [9,10]. High quality, well-designed research that aligns with practice is greatly needed to build a pragmatic evidence base for manipulative therapy.

Thus, the Chuna Research Network (CRN), consisting of two universities, two spine-specialty hospitals, and the Korea Institute of Oriental medicine (KIOM), determined a sufficiently powered sample (*n* = 194) through a pilot trial [11]. We conducted a full-scale pragmatic, confirmative RCT evaluating the comparative effectiveness and safety of CMT for non-acute LBP including an economic evaluation. We report on the effectiveness and safety of CMT in this paper and the results of economic analysis will be reported in a separate paper.

## 2. Experimental Section

### 2.1. Study Design and Setting

A parallel, two-armed, multi-center, assessor blinded, pragmatic RCT was conducted at two universities of Korean medicine hospitals (Pusan National University, Kyung Hee University at Gangdong) and two spine-specialty hospitals (Jaseng Hospital of Korean Medicine and Mokhuri Neck and Back Hospital) from 20 March 2017 to 24 January 2018 in Korea. We comprised a CRN to facilitate collaborative research between the four Korean medical institutions and the KIOM. Multidisciplinary expert discussions (encompassing experts in rehabilitation medicine, clinical research and economic analysis, a statistician, and a clinical research organization (CRO)) were carried out in face-to-face meetings to review the process, methods, and results once a month for the duration of the trial period.

The trial was conducted with participants’ written informed consent and institutional review board (IRB) approval (approval date: Pusan National University Korean Medicine Hospital on 26 December 2016). Periodic monitoring and data management were performed regularly by the contracted CRO.

**Figure 1:**
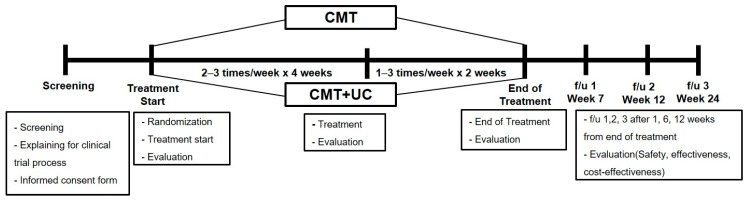
Research process of the study. CMT: Chuna manipulative therapy; UC: usual care; f/u: follow-up.

The details of the research process are summarized in Table S1 and Figure 1 shows a short guide of the research process. Further information is available at the clinical trial registration site Clinical Research Information Service (CRIS, https://cris.nih.go.kr; KCT0002329) and in the previous pilot study [11].

### 2.2. Participants

Advertisements were used to recruit target subjects. As several departments/clinics in the hospital were involved in the treatment of LBP, advertisements were used in the hospital to recruit the target subjects. Physicians prospectively identified patients at their clinics and consulted possible patients with LBP to the principal investigator (PI). The PI encouraged them to take part in the trial. Those interested in the trial met the clinical research coordinator (CRC), who confirmed eligibility by collecting further data. We selected participants who met eligibility and received written informed consent. Subjects were included if they met the following criteria.

#### 2.2.1. Inclusion Criteria

Subjects who were aged 19 to 70 years old, with non-acute LBP (≥ 3 weeks duration, subacute or chronic) and an average numerical rating scale (NRS) of ≥ 5 during the past week, who agreed to trial participation and provided written informed consent, were included regardless of the presence or absence of leg pain.

#### 2.2.2. Exclusion Criteria

We excluded patients for the following reasons: (1) diagnosis of serious pathologies which might cause LBP (e.g., spinal dislocation, acute fracture, spinal metastasis of tumors); (2) suspected fracture according to the researcher’s clinical judgment; (3) recent spinal surgery history within the previous three months; (4) other chronic diseases which might interfere with the effect of treatment or interpretation of outcomes (e.g., chronic renal failure); (5) progressive neurologic deficits or severe neurologic symptoms such as cauda equina syndrome; (6) inner fixation or stabilization devices applied by spinal surgery; (7) current treatment with steroids or immune-suppressants; (8) medications for mental illness or other medications that could interfere with study results; (9) previous CMT experience; (10) medication such as non-steroidal anti-inflammatory drugs (NSAIDs) or invasive treatments such as acupuncture or injections within the past week; (11) pregnancy, breastfeeding, or plans of pregnancy; and (12) participation in other clinical studies or unsuitability for other reasons as deemed by the researchers.

Participants who received hospitalization, surgery, procedures, or medication that could interfere with the results of the study during the treatment period (6 weeks) were excluded without consultation with the researchers.

### 2.3. Randomization, Allocation Concealment

An independent statistician generated the random allocation sequence using SAS 9.3 (SAS Institute Inc., Cary, NC, USA) and provided it to Pusan National University Korean Medicine Hospital, the principal medical institution of the CRN. The principal research institute assigned the same number of patients to each of the two groups by block randomization assignment. The block size (*n* = 4) was not known to the personnel who were recruiting patients or performing patient allocation. Following screening, participants who met the inclusion criteria were randomly allocated into two groups (CMT + usual care (UC) or UC alone) at a ratio of 1:1. Allocation was concealed by central allocation.

### 2.4. Blinding

Due to the dissimilarity of interventions between groups, it was impossible for physicians and participants allocated to the treatment groups to be blinded. Only outcome assessors, the statistician, and data analysts were blinded, and physicians who did not engage in the treatment interventions conducted outcome assessments in a separate room after treatment. We also cautioned participants not to inform the outcome assessor of treatment allocation prior to each assessment. The electronic data sets that did not contain information on group allocation were sent to independent statisticians and data analysts.

### 2.5. Sample Size

Based on previous literature and our pilot study, the following assumptions were made when determining our sample size: (1) level of significance α = 0.05, (2) type 2 error β = 0.2, with test power set at 80%, and (3) reference to outcomes from a prior pilot trial [11] which used the NRS as the primary measure. Mean difference and standard deviation (SD) between the two groups were respectively specified as 1.5 [12] and 3.3 based on an intention-to-treat (ITT) analysis by a statistician, and (4) estimated compliance was set as 80%. We used nQuery Advisor 7.0 to calculate target count; 77 people per group were required as the minimum number to prove the above assumptions. The goal was to recruit a total of 194 participants with 97 per group, taking into account a 20% non-compliance rate.

### 2.6. Interventions

The subjects were treated for six consecutive weeks with either CMT plus UC or UC alone. Additional treatments (e.g., procedures, acupuncture, or surgery) were not allowed during the 6-week period of intervention. Orally administered medicines for hypertension and diabetes were permitted and recorded in detail to compare the difference between groups at the end of the study. Cases receiving injections such as steroid nerve blocks were monitored and regarded as dropouts. Details on the Chuna techniques and UC used in the previous trial and detailed instruction materials utilized in this subsequent full-scale study can be found in the pilot protocol [13], and pilot trial [11].

All physicians involved in the study received education on the predetermined protocol, SOP (Standard Operating Procedures), Helsinki Declaration, and Korean Good Clinical Practice Guidelines for the protection of study participants.

#### 2.6.1. Chuna Manipulative Therapy

CMT focuses on meridian theory and meridian muscle concepts, and CMT clinicians also focus the breathing of patients during the treatment procedure. CMT adopted a semi-standardized treatment plan established by selecting Chuna technique based on CMT expert opinions on LBP collected via surveys of Chuna clinical experts (total *n* = 20) [13]. A survey for standardization was conducted to find the validity of the duration of treatment session and adequate technique. After the survey, experts (from Korean medical rehabilitation medicine with two clinical research methodologists and a subcommittee for standardization of seven specialists) held two discussion meetings and reviewed and agreed upon decision making using the physician’s judgement [14]. The physicians administering CMT in this trial were Korean medical doctors with more than three years of clinical experience using CMT, and received CMT protocol training sessions (two sessions, 4 h/session) for standardized applications. The CMT techniques utilized in this study were divided into lumbar and pelvic regions, and mandatory or selective techniques were performed following physician judgement [13]. CMT sessions were administered over a period of 6 weeks. Whether compliance was 80% or higher was assessed at the last visit of week 4 and the last visit of week 6 which marked the end of treatment. The frequency of treatment sessions allowed for a difference by period, at 2–3 sessions/week in week 1 to week 4 and 1–3 sessions/week in week 5 to week 6 based on CMT physicians’ judgement regarding results of previous treatment sessions and to reflect actual clinical practice conditions. A total of 10 to 18 CMT sessions (at least or more than the 10 sessions that participants need to receive) were administered over a 6-week period. The time duration of one CMT session consisted of approximately 5 min of diagnosis and 10 min of treatment. The modalities we utilized were shown in a previous pilot protocol, and this gave additional information [13].

#### 2.6.2. Usual Care

UC was composed of physical therapy and back pain education in this study. Pain education was allowed according to subjects’ pain intensity, but monitored to compare the difference between groups. Physical therapy of UC was provided with reference to a list of the most frequently used treatments ranked from 1 to 10 in LBP patients compiled from the 2011 Korean Health Insurance Review and Assessment (HIRA) statistics [15]. Two of the top ten were selected by the clinicians and implemented for 10 min each. For blinding purposes of the outcome assessor, frequency and type of physical therapy used were recorded in a separate case report form. The physical therapy frequency of treatment sessions allowed for a difference by period, at 2–3 sessions/week in week 1 to week 4 and 1–3 sessions/week in week 5 to week 6 based on physicians’ judgement, considering the results of previous treatment sessions and real clinical conditions. Back pain education was provided to participants of both groups equally with standardized presentation after enrollment for duration of about 15 min. A standardized face-to-face education presentation after registration provided equal back pain training to participants of both groups. The structured education program explained the physiology, pathology, and epidemiology of LBP and was also delivered in brochure format.

### 2.7. Outcomes

#### 2.7.1. Primary Outcome Measurement

The primary outcome was LBP graded on an NRS which quantifies the subjective pain experienced by patients over the past week [16,17]. The patient selects the number that most closely reflects their pain intensity, considering 0 to be without pain and 10 to be extreme pain, with higher values indicating greater pain. There is a general consensus that the NRS has greater validity and strength than other scales [18]. The Pearson correlation coefficient comparing NRS and visual analogue scale (VAS) shows very strong validity and reliability (*r* = 0.93) [2,19].

#### 2.7.2. Secondary Outcome Measures

Secondary outcome measures included leg pain level for the past week as assessed with the NRS. Functional status of LBP was assessed by using the Korean version of the Oswestry Disability Index (ODI) questionnaire [20]. Each item has a six-level answer choice (with corresponding scores of 0–5), with a total score of 50. Higher scores reflect greater disability. The correlation coefficient comparing the ODI and VAS of LBP (*r* = 0.38) shows moderate validity [21]; however, it is reliable (effect size (ES), (0.65) [22], Cronbach’s α (0.7)) [21]. The patient global impression of change (PGIC), initially developed for psychological use [23], was used to assess comprehensive and global change in LBP and movement limitations due to pain [24]. The PGIC consists of answers ranging from 1 to 7 with lower numbers indicating higher treatment. The EuroQol-5 dimension (EQ-5D) is a measurement tool composed of five dimensions assessing current health status, which consists of mobility, self-care, usual activities, pain/discomfort, and anxiety/depression. The EQ-5D is a widely used tool for measuring health-related quality of life across the health care sector. Each dimension was evaluated using three-level answers, with lower scores reflecting a better health status of the patients [24]. Correlation between EQ-5D and NRS (*r* = 0.67) [25] shows strong validity, with a reliable ES (0.53) [26]. The Health Utility Index III (HUI-III) was used to calculate participants’ quality of life in addition to EQ-5D [27]. Lumbar range of movement (ROM) was used to objectively assess functional improvements after treatment [28]. While ROM evaluation is valid (*r* = 0.97) and reliable (*r* = 0.94) [29], it is not highly responsive (ES 0.1–0.6) [30,31]. The angle between a perpendicular line and the patient’s lumbar spine was measured using a goniometer at maximum lumbar flexion, extension, side bending, and rotation of each side. If measurement was not possible due to pain, the angle was recorded as 0°.

### 2.8. Statistical Analysis

ITT analysis was the primary analysis method and the last observation carried forward (LOCF) method was used. Additional analyses were performed in subjects who completed clinical trial participation, excluding dropouts. The primary endpoint was at 7 weeks from commencement of treatment after random allocation.

Data were summarized using descriptive statistics: frequency (percentage for categorical variables and mean ± SD for continuous variables). Differences in study participants’ characteristics were compared across subgroups with the chi-squared test or Fisher’s exact test for categorical variables and analysis of variance, as appropriate. In the case of continuous variables, independent *t*-test was performed if normality was satisfied. If not, Wilcoxon rank sum test was performed. Paired *t*-tests and independent *t*-tests were employed to assess differences between assessment points or the two groups. Normality was tested with the Shapiro–Wilk test. To reduce error due to inequality of groups at baseline, analysis of covariance (ANCOVA) was employed, using the baseline value as a covariate. An independent statistical expert performed the analyses using SPSS for Windows 22.0 statistical software (IBM Corp. Armonk, NY, USA). All tests were two-tailed with a 5% significance level. ES was calculated with the G *Power V.3.1.9.2 program for Windows (Heinrich-Heine-Universität, Düsseldorf, Germany).

### 2.9. Safety

To monitor safety of CMT and UC, participants were asked about adverse events (AEs) at every visit. If AEs occurred, physicians rated the relationship between each treatment using a six-point scale (1 = definitely related; 2 = probably not related; 3 = possibly related; 4 = probably not related; 5 = definitely not related; and 6 = unknown) and categorized them into three levels (mild, moderate, and severe). If serious adverse events (SAEs) occurred during the study, unblinding was allowed and the researcher would inform the relevant IRB and main study site (Pusan National University Korean Medicine Hospital) to decide whether the trial would be continued or prematurely terminated. Participants who suffered AEs received appropriate medical action and damage compensation.

### 2.10. Availability of Data and Material

The datasets generated and/or analyzed during the current study are not publicly available due to conditions of ethical approval but are available from the corresponding author on reasonable request.

## 3. Results

### 3.1. Patient Characteristics

**Figure 2:**
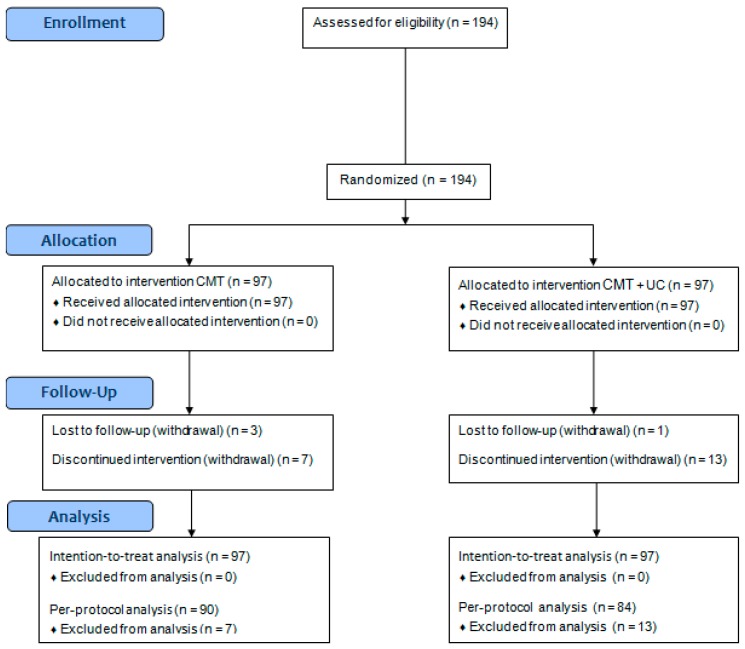
Consolidated Standards of Reporting Trials (CONSORT) 2010 flow diagram. CMT: Chuna manipulative therapy; UC: usual care.

Figure 2 shows the Consolidated Standards of Reporting Trials (CONSORT) [32] diagram including participant numbers for enrollment, allocation, follow-up, and analysis. A total of 194 participants were eligible and allocated to the two groups at four medical institutions. Twenty patients dropped out during the treatment period due to consent withdrawal and four patients dropped out after finishing all treatments because they were lost to follow up (drop-out rate: 12.37%). A total of 194 participants were subjected to the ITT analysis, and 174 participants (CMT + UC: 90, UC: 84) were included in the per-protocol analysis. The trial results are reported with ITT analysis. Table 1 shows the demographic and health features of the participants at baseline (week 1). There were no statistically significant differences between the two groups at baseline except for age and alcohol consumption. The averages of total visits during the treatment period (6 weeks) showed no statistically significant differences between groups (CMT + UC: 11.14 ± 3.26, UC: 11.04 ± 4.06, *p* = 0.845).

### 3.2. Primary Outcome

**Figure 3:**
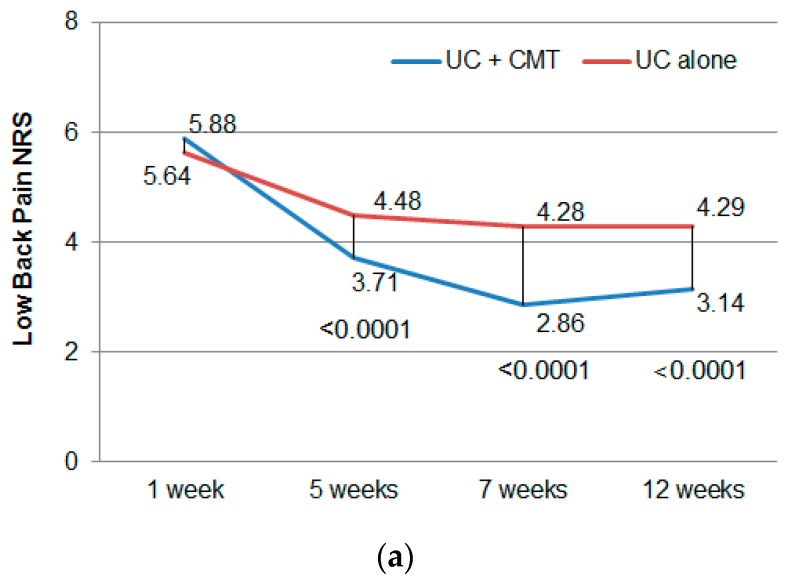

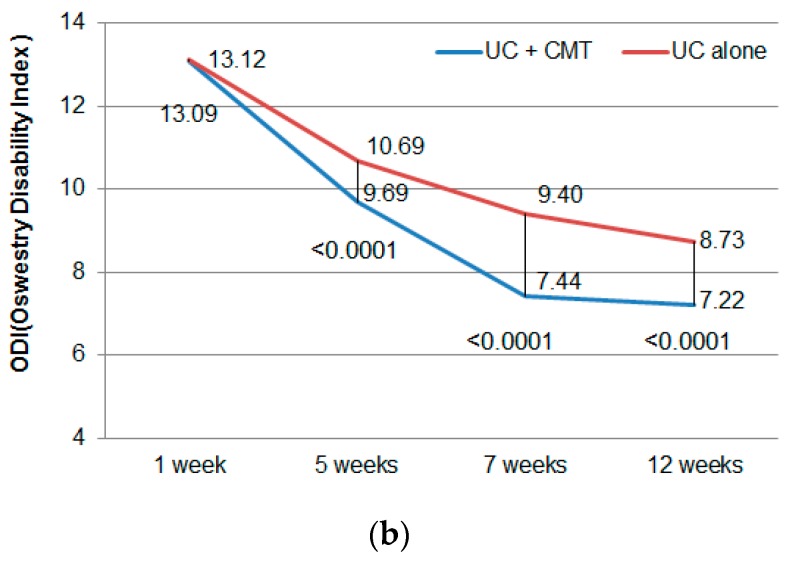
The changes in pain intensity of lower back pain and function by the Oswestry Disability Index at each endpoint. (**a**) The mean numerical rating scale (NRS) of lower back pain from baseline through 12 weeks of follow-up. Follow-up data were collected at 5, 7, and 12 weeks. (**b**) The mean Oswestry Disability Index (ODI) scores from baseline (1 week) through 12 weeks of follow-up. Follow-up data were collected at 5, 7, and 12 weeks. CMT: Chuna manipulative therapy; UC: usual care.

In Figure 3a, the NRS of LBP showed a statistically significant improvement at the primary endpoint (week 7) compared to week-1 data both between groups (CMT + UC: −3.02 ± 1.72, UC: −1.36 ± 1.75, *p* < 0.0001) and within groups (*p* < 0.0001).

### 3.3. Secondary Outcome

Figure 3b shows a significant decrease in mean ODI scores from baseline (1 week) through the 12-week follow-up period.

Table 2 shows a statistically significant improvement of the following measurements between groups at seven weeks: ODI scores (CMT + UC: −5.65 ± 4.29, UC: −3.72 ± 4.63, *p* = 0.003), NRS of leg pain (CMT + UC: −2.00 ± 2.33, UC: −0.44 ± 1.86, *p* < 0.0001), PGIC (CMT + UC: −0.28 ± 0.85, UC: 0.01 ± 0.66, *p* = 0.0119), and ROM (Right rotation, CMT + UC: 2.42 ± 8.86, UC: -0.35±7.78, *p* = 0.0289; Left rotation, CMT + UC: 3.02 ± 8.56, UC: 0.23 ± 8.47, *p* = 0.0307) at 7 weeks, showing a statistically significant improvement between groups.

### 3.4. Adverse Events

AEs of minor to moderate severity in 21 subjects were reported during the trial and there were no significant differences between the two groups in frequency of AEs in the two groups (Table S2). Most of the reported AEs resolved after a certain period of time, and there were no cases of dropout or code breaking due to AEs. Cases of AEs during the trial period were not highly related to the treatments.

## 4. Discussion

CMT is a Korean type of manipulative therapy which is used widely for managing LBP in South Korea, with high patient satisfaction [3,33]. CMT has long history and comprises a different system of theory compared to other spinal manipulative therapies [9]. Actually, the difference in history and theory does not indicate a difference in effectiveness. Our results revealed statistically and clinically significant improvement in outcomes of pain, function, and quality of life in patients with LBP at the end of CMT at six months mid-term follow-up compared to UC alone.

Research about manipulative therapy has existed over the past decade. While the updated Cochrane review on spinal manipulative therapies for chronic LBP covers many study designs similar to the present study comparing spinal manipulation with other treatments such as UC, physiotherapy, exercise, medical care, or as an add-on treatment to other treatments, the evidence for the effect of spinal manipulative therapy is inconclusive and insufficient; methodologically well-conducted research remains rare. Studies need to be replicated in diverse settings to obtain sufficient evidence [34]. Research examining the comparative effectiveness of manipulative therapy for LBP found no significant difference in benefit when compared with standard allopathic treatment [35], group exercise, or physiotherapy [36], or sham manipulation [37]. The high level of heterogeneity across the previous studies significantly limits our ability to draw firm conclusions about the comparative effectiveness to the stakeholder of the health service [34]. Under these circumstances it is necessary to search for solutions to increase the accessibility of medical services to CMT and to improve patient health and quality of life [38,39]. It is important to determine the comparative effectiveness and safety of CMT. Previous Cochrane reviews highlighted the urgent need for research on cost-effectiveness studies [3]. Although we did not publish the economic evaluation at the same time in this paper, our economic evaluation will be reported in separate paper.

A study reported that successful average NRS changes (2.66) in lumbar spine surgery patients are well above the minimal clinically important difference (MCID) range (0.28–4.5) [40,41,42,43,44]. The NRS difference in this study from baseline to 7 weeks was 3.02, which is a meaningful value compared with other studies and is in the MCID range. The ES of the primary outcome of this study measured at 7 weeks was large (0.96), which is noteworthy. The ESs of primary outcomes at 1 month were 0.22, 0.28 [45,46] in a study selecting sham manipulative therapy as a control and 0.35 [46] in a study selecting manipulative method plus UC as a control, similarly to our design. In particular, the ESs of outcome with various interventions were 0.03–0.57 at 1 month and 0.06–0.79 at 2 months [3]. A major strength of this study was that we adopted a pragmatic study design which enhanced external validity [47] and chose treatment interventions based on clinical expert opinions. Therefore, we think CMT can be used widely considering that this study reflects real clinical conditions as practiced in Korea.

Some limitations of our study are attributable to the fact that CMT entails contact with patients when treating, rendering it impossible to blind patients due to its method of treatment. Potential bias is a possible liability in this study design, but is difficult to control due to unblinding [3,48]. Experts considered sham control in meetings, but as CMT would generally be provided for an average of 15 min per patient, it was concluded that a sham-controlled trial would be unrealistic in Korean clinical settings. Even though actual contact generates small amount of effect, it may stimulate cognitive effect [49]. Selecting an appropriate placebo for an RCT of manipulative therapy is potentially difficult and remains questionable. Regarding a suitable placebo for a trial of manipulative therapy, no consensus has existed among experts, including both clinicians and academics. Of the pragmatic RCTs of manipulative therapy, we found two studies of manipulative therapy included a placebo intervention for chronic LBP [45,50]. We carefully note that gap between ESs mentioned above could imply the placebo effect by unblinding. Concerns about which trial design is more valuable with respect to manipulative therapy have existed. The aims of the two designs are different. RCTs with placebo are able to test the efficacy of manipulative therapy, but a pragmatic trial can evaluate the effectiveness of everyday clinical practice [51,52]. Therefore, we adopted a pragmatic RCT design [47] to apply the result in the real world setting.

Future trials may consider other conventional treatments (e.g., orally administered medicine) or other treatments (e.g., sham CMT, active exercise with instructor, acupuncture etc.) in a control group with large sample size and pragmatic design. Observing the long-term impact of CMT would be required considering that our study used a 6-month mid-term follow-up. In extended research, various subgroup analyses based on sex and age may be considered for evidence of spinal manipulative therapy. There is a need for researchers to systematically collect and report the details of manipulative therapy in comparative effectiveness studies to ensure findings can inform policy and practice.

## 5. Conclusions

This study was the first well-designed RCT to show the comparative effectiveness and safety of CMT plus UC in achieving better outcomes in pain reduction, functional improvement, and higher quality of life compared to UC alone for managing non-acute LBP in conditions reflecting the real clinical settings of Korea. Our findings emphasize the importance of CMT in rigorous human clinical trials for patient health benefits, for consideration in the health insurance policy in Korea.

## Figures and Tables

**Table 1:** Descriptive statistics of demographic and health status variables at baseline (1 week)

Characteristic	Usual Care + CMT (*n* = 97) *n* (%) or Mean (SD)	Usual Care (*n* = 97) *n* (%) or Mean (SD)
Sex (male)	17 (17.5)	23 (23.7)
Age (years)	44.5 ± 12.5	39.0 ± 11.6
Height (cm)	162.5 ± 7.5	164.6 ± 7.7
Weight (kg)	62.3 ± 11.4	61.5 ± 13.0
BMI (kg/m)	23.47 ± 3.24	22.58 ± 3.67
Smoking		
Non-smoker	87 (89.7)	82 (85.4)
Ex-smoker	8 (8.3)	10 (10.4)
Smoker	2 (2.1)	4 (4.2)
Alcohol consumption		
No	67 (69.1)	53 (54.6)
Yes	30 (30.9)	44 (45.4)
Duration LBP (years)	6.45 ± 6.66	4.85 ± 5.10
**Primary outcome**		
NRS of lower back pain	5.88 ± 0.89	5.64 ± 1.00
**Secondary outcomes**		
NRS of radiating leg pain	4.18 ± 2.38	3.51 ± 2.26
PGIC (5 weeks)	2.24 ± 0.84	2.95 ± 0.80
ODI	13.09 ± 4.41	13.12 ± 5.22
ODI (%)	26.19 ± 8.81	26.25 ± 10.45
ROM (Flexion)	85.15 ± 18.24	86.96 ± 16.37
ROM (Extension)	18.14 ± 5.74	19.69 ± 6.61
ROM (Lateroflexion Rt.)	24.18 ± 7.10	24.8 ± 5.93
ROM (Lateroflexion Lt.)	24.69 ± 6.80	25.37 ± 5.64
ROM (Rotation Rt.)	39.79 ± 11.51	41.44 ± 10.26
ROM (Rotation Lt.)	39.53 ± 11.68	41.7 ± 11.13
EQ-5D	0.84 ± 0.09	0.85 ± 0.09

All values are mean ± standard deviation except sex, smoking, and alcohol consumption. CMT: Chuna manipulative therapy; SD: standard deviation; BMI: body mass index; NRS: numeric rating scale; PGIC: patient global impression of change; ODI: Oswestry Disability Index; ROM: range of motion; Rt.: Right; Lt.: Left; EQ-5D: EuroQol-5 dimension.

**Table 2:** Mean differences for usual care alone versus usual care plus CMT on primary outcome secondary outcome variables at each assessment point.

	UC+CMT (*n* = 97) Mean ± SD	UC (*n* = 97) Mean ± SD	*p*-Value
**NRS (LBP)**			
Baseline	5.88 ± 0.89	5.64 ± 1.00	
7th week	2.86 ± 1.84	4.28 ± 1.75	
Difference	−3.02 ± 1.72	−1.36 ± 1.75	<0.0001 ^(1)^
12th week	3.14 ± 2.09	4.29 ± 1.96	
Difference	−2.73 ± 2	−1.35 ± 1.9	<0.0001 ^(2)^
24th week	3.44 ± 2.18	4.52 ± 1.92	
Difference	−2.43 ± 2.09	−1.12 ± 1.76	<0.0001 ^(3)^
**Radiating pain leg NRS**			
Baseline	4.38 ± 2.24	3.68 ± 2.18	
7th week	2.29 ± 2.01	3.11 ± 2.18	
Difference	−2.00 ± 2.33	−0.44 ± 1.86	<0.0001 ^(1)^
12th week	2.46 ± 2.06	3.05 ± 2.18	
Difference	−1.78 ± 2.11	−0.44 ± 1.97	<0.0001 ^(2)^
24th week	2.64 ± 2.43	3.5 ± 2.21	
Difference	−1.67 ± 2.56	−0.07 ± 2.34	<0.0001 ^(3)^
**PGIC**			
5th week	2.24 ± 0.84	2.95 ± 0.8	
7th week	1.96 ± 0.82	2.98 ± 0.78	
Difference	−0.28 ± 0.85	0.01 ± 0.66	0.0119 ^(^^4)^
12th week	2.1 ± 0.87	2.92 ± 0.88	
Difference	−0.13 ± 1.01	−0.05 ± 0.78	0.5185 ^(^^5)^
**ODI**			
Baseline	13.09 ± 4.41	13.12 ± 5.22	
7th week	7.44 ± 5.29	9.4 ± 5.58	
Difference	−5.65 ± 4.29	−3.72 ± 4.63	0.003 ^(1)^
12th week	7.22 ± 5.36	8.73 ± 5.71	
Difference	−5.88 ± 4.42	−4.39 ± 4.78	0.0257 ^(2)^
**EQ-5D**			
Baseline	0.84 ± 0.09	0.85 ± 0.09	
7th week	0.90 ± 0.08	0.89 ± 0.07	
Difference	0.05 ± 0.07	0.04 ± 0.09	0.2056 ^(1)^
12th week	0.9 ± 0.07	0.89 ± 0.08	
Difference	0.06 ± 0.07	0.04 ± 0.1	0.0732 ^(2)^
24th week	0.91 ± 0.06	0.9 ± 0.07	
Difference	0.07 ± 0.08	0.05 ± 0.09	0.0587 ^(3)^
**ROM (Rotation Right)**			
Baseline	39.79 ± 11.51	41.44 ± 10.26	
7th week	42.45 ± 10.34	41.22 ± 10	
Difference	2.42 ± 8.86	−0.35 ± 7.78	0.0289 ^(1)^
12th week	42.3 ± 10.86	42.13 ± 10.94	
Difference	3.26 ± 9.51	−0.91 ± 9.53	0.113 ^(2)^
**ROM (Rotation Left)**			
Baseline	39.53 ± 11.68	41.7 ± 11.13	
7th week	42.88 ± 11.15	42.21 ± 11.02	
Difference	3.02 ± 8.56	0.23 ± 8.47	0.0307 ^(1)^
12th week	43.16 ± 10.68	42.74 ± 11.39	
Difference	4.13 ± 9.8	1.16 ± 9.79	0.0512 ^(2)^

All values are mean ± standard deviation. Statistical analysis was conducted based on intention-to-treat analysis with missing values imputed with last observation carries forward method (LOCF). CMT: Chuna manipulative therapy; UC: usual care; SD: standard deviation; NRS: Numerical rating scale; LBP: Lower back pain; ODI: Oswestry disability index; PGIC: patient’s global impression of change; EQ-5D: EuroQol five dimensions questionnaire; ROM: Range of motion. ^(1)^ *p*-values were derived from *t*-test for comparison of difference 7 weeks from the baseline (1 week) between groups. ^(2)^ *p*-values were derived from *t*-test for comparison of difference 12 weeks from the baseline (1 week) between groups. ^(3)^ *p*-values were derived from *t*-test for comparison of difference 24 weeks from the baseline (1 week) between groups. ^(4)^ *p*-values were derived from paired *t*-test for comparison of difference between 7 weeks and 5 weeks. ^(5)^ *p*-values were derived from paired *t*-test for comparison of difference between 12 weeks and 5 weeks.

## Supplementary Materials

The following are available online at https://www.mdpi.com/2077-0383/9/1/144/s1. Table S1: Research process of each time point. Table S2. Adverse events. Table S3. Semi-standardized treatment plan of study Chuna manipulative therapy techniques based on physician’s consensus.
