# Supplementary material for: Comparative Effectiveness of Chuna Manipulative Therapy for Non-Acute Lower Back Pain: A Multi-Center, Pragmatic, Randomized Controlled Trial
Source: J Clin Med. 2020 Jan 5;9(1):144. doi: 10.3390/jcm9010144 (PMC7019562; doi:10.3390/jcm9010144)
Supplement: Supplementary file 1 [file jcm-09-00144-s001.pdf]

Table S1. Research process of each time point

Time point	Screening	Active Treatment post-allocation						Follow-up		
		Week 1	Week 2	Week 3	Week 4	Week 5	Week 6	Week 7 f/u 1	Week 12 f/u 2	Week 24 f/u 3
Informed consent form	○									
Vital sign check	○							○	○	
Socio-demographic characteristics, medical history (e.g. LBP, medication history)	○									
Physical examination	○									
Randomized allocation		○								
Treatment in UC[a], CMT+UC[a] group (times/week)		←2-3 times /week→				← 1-3 times /week→				
Symptoms and change in medicine		○	○	○	○	○	○	○	○	
NRS of LBP and leg pain	○	○	○	○	○	○	○	○	○	○
ODI		○				○		○	○	
PGIC						○		○	○	
ROM		○				○		○	○	
EQ-5D		○				○		○	○	○
HUI-Ⅲ		○				○		○	○	○
Economic evaluation		○				○		○	○	○
Adverse events		← every visit →						○	○	○

a: medication will be administered daily and physical therapy will be applied 2-3 times/week

CMT, Chuna manual therapy; UC, usual care; NRS, numeric rating scale; LBP, lower back pain; ODI, Oswestry disability index; PGIC, patient global impression of change; ROM, range of movement; EQ-5D, EuroQol-5 dimension; HUI-Ⅲ, health utility index Ⅲ

Table S2. Adverse events

Cough cold
Sore throat
Bronchitis
Acute laryngopharyngitis
Bronchiolitis
Acute bronchitis
Sputum
Runny nose
Right ear pain, oozing
Chilling
Headache
Whole body myalgia
Neck, shoulder pain
Cervical sprain
Trapezius Myalgia
Left knee pain
Hand-foot numbness
Ankle sprain
Helicobacter pylori gastritis
Dyspepsia
Diarrhea,
Psoriasis
Itching
Laceration of the left 4th finger
Laceration of the right toe
Tinea pedis
Menstrual irregularity
Nasal plastic surgery

Table S3. Semi-standardized treatment plan of study Chuna manipulative therapy techniques based on physician’s consensus

Region	Mandatory techniques	Selective techniques
Lumbar spine[a]	• distraction technique<br>Spine flexion distraction technique (flexion)<br>• correction technique<br>Sidelying lumbar (extension or flexion) dysfunction correction technique	• distraction & mobilization technique<br>- Spinal flexion distraction (circumduction)<br>- Spinal flexion distraction (side-bending)<br>• meridian muscle technique<br>-Iliopsoas myofascial *Chuna*
Pelvis[a]	<Iliac dysfunction (if applicable by evaluation) ><br>• correction technique<br>- Prone iliac anterior rotation dysfunction correction technique/ or<br>- Prone iliac posterior rotation/sacral sidebending dysfunction correction technique<br>- Prone leg raise iliac dysfunction correction technique<br>- Prone inflare-outflare dysfunction correction technique<br><br>< Sacral dysfunction (if applicable by evaluation) ><br>• correction technique<br>- Prone sacral side-bending and rotation dysfunction correction technique	• Prone sacral dysfunction correction technique (extension or flexion)

a Dysfunction type assessed and diagnosed prior to treatment based on the textbook of [Chuna manual medicine 2.5 ed, 20176]

-General process consisted of diagnosis (about 5 min), treatment and recheck (about 10 min).

-Chuna manipulative technique is based on Korean medicine diagnosis & treatment include meridian, meridian muscle, and Do-in exercise.
